# Risk, Responsibility and Surgery in the 1890s and Early 1900s

**DOI:** 10.1017/mdh.2013.16

**Published:** 2013-07

**Authors:** Claire Brock

**Affiliations:** School of English, University of Leicester, University Road, Leicester LE1 7RH, UK

**Keywords:** Surgery, Risk, Negligence, Responsibility, 1890s–1900s, Women Surgeons

## Abstract

This article explores the ways in which risk and responsibility were conceptualised in the late nineteenth and early twentieth centuries by surgeons, their patients and the lay public. By this point surgery could be seen, simultaneously, as safe (due to developments in surgical science) and increasingly risky (because such progress allowed for greater experimentation). With the glorification of the heroic surgeon in the late Victorian and early Edwardian period came a corresponding, if grudging, recognition that successful surgery was supported by a team of ancillary professionals. In theory, therefore, blame for mistakes could be shared amongst the team; in practice, this was not always the case. Opening with an examination of the May Thorne negligence case of 1904, I will also, in the latter third of this piece, focus on surgical risks encountered by women surgeons, themselves still relatively new and, therefore, potentially risky individuals. A brief case study of the ways in which one female-run institution, the New Hospital for Women, dealt with debates surrounding risk and responsibility concludes this article. The origin of the risks perceived and the ways in which responsibility was taken (or not) for risky procedures will provide ways of conceptualising what ‘surgical anxiety’ meant in the 1890s and 1900s.

In early June 1904, Dr May Thorne was tried for alleged negligence. A year earlier, she had supposedly left a sponge or swab inside a patient, Miss Byrne, upon whom she had performed an abdominal operation. At the core of the accusation of neglect was Thorne’s alleged failure to count the sponges employed during an operation which had taken place in a private nursing home run by Thorne’s only assistant, qualified nurse, Mrs Palmer. There was no doubt that May Thorne had performed a difficult and skilful procedure, removing a large abscess which had been adherent both to Miss Byrne’s uterus and to one of her fallopian tubes. The trial hinged on the question of responsibility, with the judge directing the jury to answer five key questions during their deliberations. First, was the defendant guilty of a want of due and reasonable care in the counting or superintending the counting of the sponges? Second, was Mrs Palmer employed by the defendant as an assistant during the operation? Third, was Mrs Palmer negligent in counting the sponges? Fourth, was the counting of the sponges a vital part of the operation which the defendant undertook to see properly performed? And, finally, was Mrs Palmer under the control of the defendant during the operation? The jury returned positive responses to all five questions, awarding damages of a farthing to Miss Byrne because Thorne had performed the operation without fee, and £25 for resulting pain and suffering.^1^ On 6 June May Thorne left the court with her reputation as a skilful surgeon intact, but with a query over her management and, by implication, her profession’s management both of surgical personnel and the operating theatre.

An ‘Historical Perspective’ on ‘Risk and Medical Innovation’, according to Thomas Schlich, ‘provides the background information for finding appropriate strategies for coping with the uncertainties surrounding potential threats to health, whether they are seen as originating in the environment, individual behaviour or in medicine itself’.^2^ This article will use May Thorne’s trial as a starting point to consider the ways in which risk and responsibility were conceptualised in the late nineteenth and early twentieth centuries by professionals, their patients and the lay public. I will focus in the latter third upon surgical risks encountered by women as patients, but also as surgeons, the latter still relatively new and, therefore, potentially risky individuals. In doing so, I will examine how women surgeons operated in private, as in the case of May Thorne, and in an institutional setting, the New Hospital for Women, where Thorne was employed as house surgeon and anaesthetist in the early 1900s.^3^ At the turn of the twentieth century, according to late Victorian and early Edwardian surgeons, surgery could be seen simultaneously as possessing ‘so little risk’ and surrounded with ‘special anxieties’.^4^ The origin of the risks perceived and the ways in which responsibility was taken (or not) for risky procedures will provide ways of conceptualising what ‘surgical anxiety’ meant in the 1890s and 1900s.

## Byrne versus Thorne

It is worth looking in closer detail at the Thorne trial because of the specific questions it raised about the practice of surgery in the early twentieth century and the complex relationships which had developed between the surgeon and surgical personnel. As Christopher Lawrence has noted, Edwardian surgery can be characterised both by the cult of the heroic surgeon, embodied by Joseph Lister, and the grudging recognition that surgeons were now supported by a skilled team within the operating theatre and without.^5^ In the 1890s and early 1900s the promotion of individual surgical skill jostled uncomfortably with the crediting of the wider team – anaesthetists, pathologists, bacteriologists, physiologists, trained nurses – who provided vital support. While historians of medicine have commented recently on the building up of trust between the surgeon, the surgical team and the patient at this point, May Thorne’s trial and the reaction to it suggested that doubt and uncertainty caused fissures in this fragile three-way relationship.^6^

In April 1903 Miss Byrne, who was employed as a housekeeper, consulted Dr May Thorne at her private London practice.^7^ The nature of the case was such that Thorne recommended an operation for Miss Byrne’s life-threateningly large pelvic tumour which, she suggested, should be undergone in a nursing home owned by a Mrs Palmer. Thorne herself performed the operation and it was a success. However, Miss Byrne soon experienced discomfort and, as she was then in Brighton, she consulted a local doctor named Calvert, who advised that she must have another operation. This procedure was duly carried out at the Sussex Hospital. When Miss Byrne was opened up, it was discovered that an abscess had formed due to the presence of a mattress sponge which had clearly been left inside the patient after the first operation. Rapid recovery ensued, along with Miss Byrne’s swift determination to recover the expenses for her second procedure, as well as damages for alleged negligence by her original surgeon.

Despite the submission of May Thorne’s counsel, Mr Dickens, that there was no evidence for negligent behaviour, the judge concluded that a jury must decide and the case proceeded to trial. Neglect was attributed to May Thorne because of her alleged failure to count the sponges employed during the operation. The doctor who had removed the sponge from the patient, Calvert, claimed that sponges should be counted both before and after the operation by surgeon and nurse. While many accepted the nurse’s assistance in this necessary procedure, Calvert asserted that the surgeon should also be responsible for the accounting of surgical paraphernalia. Thorne had, by contrast, allowed her nurse to tidy up; her attention was focused, as was only right, on the operation itself and the condition of the patient. How could May Thorne, claimed her counsel, be responsible for the actions of another?

When Dr Thorne took the stand herself, she elaborated upon the background to the case. Aware that not all could afford to pay medical fees, Thorne held what she labelled a ‘cheap day’ for poor patients and it was this particular day of the week when she encountered Miss Byrne. Later consulting with her former surgical colleague at the New Hospital for Women, Louisa Aldrich-Blake, the decision was made to operate upon the supposed tumour. Confident in both the nursing home and Mrs Palmer’s skilled assistance, Thorne went ahead, her surroundings prepared as they always were when she used Mrs Palmer’s facilities. When she was opened up, it was discovered that Miss Byrne had an abscess which Dr Thorne successfully removed. Utilising twenty-six sponges in all – twenty-four swabs and two large mattresses – Thorne ensured that Mrs Palmer remained in charge of their distribution and, after double checking with Mrs Palmer that the number was correct, closed the abdomen. For May Thorne, the counting of sponges was the responsibility of the attendant nurse. While she herself had checked her instruments, Mrs Palmer had been in charge of the sponges. And, while Thorne had removed as many as she could see during the operation, they were almost impossible to see by the surgeon in a wet and bloodied abdomen.

Medical witnesses for Dr Thorne supported her decision to assign sponge counting to an attendant assistant or nurse. Dr W.S.A. Griffith testified to the difficulty of the operation performed and noted that it could only be in the patient’s interests to delegate responsibility for ancillary matters to others, while the surgeon carried out the professional, ‘vital parts of the operation’.^8^ Dr Walter Tate, having performed around 600 abdominal procedures of his own, had never counted any of his own sponges, preferring to leave the task to his nurses. Both Griffith and Tate acknowledged the serious consequences for the patient of, as the latter put it, ‘having a sponge left behind’, but both insisted upon the justification of allowing the attending nurse to take responsibility for the counting and maintenance of equipment.^9^ In this particular case, Mrs Palmer, Dr Thorne’s nurse and the owner of the nursing home insisted that her count had been as thorough as always, that she even wrote down the number of sponges and was positive that she had counted out and returned the same total after the operation.

The jury’s reluctant decision to award damages to Miss Byrne reflected the complexity of the trial. Providing only positive responses to the judge’s questions about surgical responsibility, they found May Thorne guilty of neglecting her duty to her nurse and to her patient, both of whom were effectively under her charge. A derisory farthing was offered in compensation, however, because, they felt, the operation had been ‘very skilfully performed’.^10^ After retiring twice more, on the insistence of the judge, who pointed out their inconsistent approach, they settled finally on £25. Both judge and jury commented again on the skilfulness shown by May Thorne in carrying out the initial operation. In spite of the focus on surgical skill, the prosecution had attempted to turn the profession against itself by quoting from a popular textbook, Frederick Treves’ *Manual of Operative Surgery* (1892), which labelled ‘[t]he leaving of a sponge or instrument an unfortunate lack of care’ on the surgeon’s part.^11^ As *The Lancet* noted, however, there was a careful distinction between a want of skill and a want of care: the former had certainly not been lacking. But the verdict was, effectively, ‘illogical’, as ‘if a defendant has caused injury to a plaintiff by negligence he must be answerable for the natural result of this negligence’.^12^ While the mistake was ‘avoidable’, the jury’s indecision pointed to the Thorne trial as a test case for surgical responsibility:

the supervision of the counting by the surgeon was a practical precaution the absence of which was to be regretted, but that they did not hold it to be one so imperatively called for or so indicated by custom that its omission would justify the exaction of a heavy penalty.^13^

For judge, jury and May Thorne’s professional peers, surgical skill in a risky and dangerous operation held sway over the assignment of sole and perpetual responsibility to the surgeon for errors incurred during the procedure.

## Professional Reaction and the Question of Responsibility

A member of the Medical Defence Union, May Thorne’s court expenses were guaranteed.^14^ She was, however, compelled personally to pay the damages and plaintiff’s costs, which amounted eventually to just over £200. An appeal was set up immediately by Thorne’s medical witnesses Griffith and Tate, advertised in the medical press, and the amount exceeded by nearly £20 within less than a month of the trial’s end. The excess was divided between the New Hospital for Women and the Royal Free Hospital.^15^ Evidently, the details of the Byrne versus Thorne trial made uncomfortable reading for medical practitioners. As the initial call to subscribe to the ‘Thorne Defence Fund’ noted, this was a case which had drawn the attention and ‘sympathy’ of ‘[e]very medical man engaged in surgical work’.^16^ Indeed, in the same issue of the *British Medical Journal* (*BMJ*), an editorial considered ‘The Responsibility of a Surgeon for His Assistants’ and focused on ‘certain points of interest’ which had surfaced during the Byrne versus Thorne trial of ‘importance to the medical profession at large’.^17^ The pecuniary punishment was roundly condemned, as was the judge, Mr Justice Bruce’s decision, clearly made early on in the trial, to make an example of May Thorne, in spite of the jury’s reluctance, throughout the proceedings, to defend the claims of the prosecution.

Worrying that the case opened up ‘an unpleasant vista of possibilities’, the *BMJ* feared for the ‘serious influence on operative surgery’ of making the surgeon responsible for ‘every detail, and for the act of every person engaged in or about the operating room’.^18^ The patient in the Thorne case had been operated upon successfully, *gratis*, in a complex procedure which probably would not even have been attempted only a generation ago, and was alive and well. Dr Thorne, on the other hand, was ‘mulct’ in damages and costs, despite being proclaimed ‘a most skilful and accomplished surgeon’.^19^ When she wrote to the medical periodicals to express her thanks to those who had contributed to the Thorne Defence Fund, May Thorne commended the ‘chivalrous and practical sympathy’ with which her case had been met and which had allowed her, as she put it, to ‘bear the strain and anxiety inevitable’ when one’s ‘professional character has been assailed’.^20^

Such ‘chivalry’ had not always characterised colleagues. The entry of women into the medical profession and the development of new surgical procedures overlapped in the second half of the nineteenth century. Both issues concerned medical practitioners and potential patients alike. From the very early days of women’s attempts to enter the medical profession, the possibility of them performing surgery exorcised critics. In 1859 the female surgeon had been dismissed as a mythical tautology: unimaginable, incapable and unwanted.^21^ Nearly half a century later, for once, differences between medical men and women had blurred, allowing the case of the accused female surgeon to embody the contemporary nightmares experienced by the profession itself.

The reaction of the main medical periodicals embodies the contradictory nature of contemporary surgical practice. While the surgeon was still happy to be viewed as the one whose skill and precision ensured operative success and patient survival, when it came down to the taking of responsibility for surgical risks or errors, blame had to be spread amongst the team. Thomas Schlich has discussed how control and standardisation helped to make surgery recognisably modern in the late nineteenth century and pointed to surgical instruments as one of the most basic ways in which surgeons manipulated patients’ bodies.^22^ Correspondingly, Stefan Hirschauer has explored the dynamics of the contemporary operating theatre, finding that the surgeon utilises the assistant as simply one more tool.^23^ What is interesting in the professional reaction to the Thorne case was the way in which control of even the simplest of tools was denied. Paradoxically, commentators on the trial of May Thorne sought to return surgical skill to the centre of debate while simultaneously removing the surgeon’s control of the surroundings in which the operation took place. The prosecution’s use of a specific passage on the ‘Counting of Instruments’ from Frederick Treves’ *Manual* was intriguing. The tone of the whole paragraph is passive, with no indication of intervention on the surgeon’s behalf: ‘On completing the intra-abdominal operation, great care should be taken to ensure that no sponge or instrument has been left in the depths of the cavity. The sponges and clamp forceps should be formally counted’. It concludes: ‘[t]he leaving of a sponge or instrument within the peritoneal cavity is a catastrophe which no surgeon would feel greatly disposed to make public’.^24^ This not only implies that such mistakes do happen, but also reveals the level of secrecy surrounding such incidents. There is no attribution of responsibility here at all.

Treves’ attitude to surgical instruments was apparent from the opening pages of his *Manual* and, in similar fashion to Hirschauer’s depiction of a late twentieth-century operating theatre, Treves believed assistants were vital, but essentially tools to be managed:

The assistants at an operation have an exceedingly important office to fill, and their capacity for their work must necessarily vary. It is a part of an operator’s duty to see that each assistant is fully informed of what he has to do, and, if possible, of the manner of his doing it. An unsuccessful operation is often attended by much abuse of the assistants, and by very severe criticisms of their manipulative powers. Such condemnation may be just, or may only serve to illustrate the proverb that ‘a bad workman complains of his tools’. It is during the most perplexing stages of an operation, and when things are going ill, that the indifferent operator finds that knives will not cut, that forceps will not hold, and that the clumsiness of assistants is beyond the limits of human belief.

Neither should a surgeon rely too much upon his tools. Treves recommended the personal touch, whereby fingers would always be superior to instruments in a profession which, after all, was a ‘handicraft’:

An intending subject for operation may well measure the depth of his sigh, at the sight of the surgeon, by the size of the operator’s instrument bag.

Some of the least progressive periods in the development of the surgeon’s art have been marked by the prolific production of instruments. With few exceptions, complex apparatus and appliances which are credited with being ingenious, or labour-saving, or automatic, are bad.

A great multitude of the instruments which figure in the makers’ catalogues are evidences of incompetence, and of a lack of dexterity which prevented the inventor from making full use of his hands.^25^

Treves’ attitude towards the cluttered world of surgical paraphernalia is indicative of Ghislaine Lawrence’s observation that the increase in instrumentation in the second half of the nineteenth century both amused and intrigued surgeons keen to plot the trajectory of surgical achievement through the tools of the trade.^26^ However useful instruments proved to the progress of contemporary surgery, for Treves they should only become an extension of the surgeon, rather than his replacement. While Treves contended that the ‘days of the so-called “brilliant” surgeon are over’, his championing of the individual’s skill and precision claimed otherwise.^27^

A letter written to *The Lancet* just after May Thorne’s trial would have horrified Treves, as it did the paper itself. A. Marmaduke Sheild wrote a letter about the ‘distressing calamities’ caused by ‘Sponges and Forceps Left in the Abdominal Cavity’.^28^ But, unlike other correspondents, who were keen to defend May Thorne and the profession itself, Sheild wanted to contribute an ingenious solution which would ‘render this accident impossible’. Not having experienced one of these ‘surgical disasters’, in spite of ‘operating on all sorts of abdominal cases over a series of years’, Sheild attributed his success, first, to his usage of only a few sponges and forceps, the number of which he wrote down upon a slate and, second, the verification of the numbers utilised personally. This ‘duty’ he had never left to a ‘subordinate’. Sheild had discussed the matter with his anaesthetist friend, Dr Hewitt. Hewitt’s ‘vantage’ in the operating theatre meant he could ‘judge largely of surgical practice’, which encouraged him to devise a solution for the custody of sponges:

A coarse wire tray is made containing (for myself) six compartments. This lies in the antiseptic lotion and the six sponges are each in its own compartment. At the end of the operation the tray is shown to me with each sponge in its place. The number of compartments may be made to vary with the number of sponges to be employed. Next as to pressure forceps, I am having constructed a white delf disc twelve inches in diameter and about half an inch thick. In this are bored eighteen holes equidistant round the edge. These holes are plainly numbered in red figures. The ‘clips’ stand in the disc and at the end of the operation, before the wound is closed, the operator is shown each pressure forceps in its proper place. He can see that ‘all is right’ at a glance. Added to these precautions the numbers should be written on a slate previously to operation which prevents the error of adding to the number of forceps or sponges after the operation is commenced.

This system was recommended both for hospital and private practice, with a note of caution for Sheild’s ‘professional brethren’: ‘[t]he surgeon cannot avoid personal responsibility for these serious mishaps’. Many instances of surgical mistakes had never been publicised, but the fact that they were unspoken did not mean that they had never happened. Sheild had both accepted advice and practical assistance from one of his surgical team and included them in his accounting of instruments and sponges. Ultimately, however, claimed Sheild, he was the one responsible for any errors.

In its reaction to the Thorne trial, *The Lancet* attacked Sheild’s harmless devices with particular disdain. The paper stated that the discussed ‘mechanical methods’ were put forward simply to ‘assist the surgeon in ascertaining whether the nurse’s or the house surgeon’s duties have been accurately performed’. Acknowledging that ‘the suggestion’ was ‘of importance having regard to the extended responsibility which the recent trial appears to throw upon the surgeon’, *The Lancet* concluded that ‘Mr Sheild’s views are not shared by many surgeons’. ‘All mechanical devices are liable to break down, because’, sneered the paper, ‘after all, they are served by human intelligence’.^29^ *The Lancet* chose to reinterpret Sheild’s intentions in communicating his experiences in three key ways. First, that the devices were constructed to serve as a check upon more junior members of the team in order to ensure that they were carrying out instructions carefully enough. Second, that ‘mechanisation’ was a shortcut and, therefore, acknowledged individual surgical inadequacy. Sheild’s attempt to alleviate the profession’s concerns over the issues which had come to light during the Thorne trial was effectively dismissed by *The Lancet* as futile advice for the majority of surgeons. They, of course, as May Thorne had done, relied upon their own ‘human intelligence’, skill and judgement, without resorting to artificial means of controlling the ways in which operations were performed. Finally, that the initial idea for the inventions discussed by Sheild came from the ‘vantage’ of his anaesthetist colleague rather than a fellow surgeon might explain some of the criticism aimed at those who, like Sheild, attempted the ‘labour-saving’ devices so despised by Treves.

Of all the concerns which materialised both during and after May Thorne’s trial, only one involved the patient herself, whose existence was subsumed under the weight of professional worries. Those undeserving or those who took advantage of medical and surgical care were subject to repeated criticism in the press in the late nineteenth and early twentieth centuries. By 1907, for example, it was estimated that half the population of London obtained free medical relief.^30^ Ire was usually aimed at those abusing the hospital system, but, in the case of Miss Byrne, May Thorne’s patient, the operation had been performed both privately and without cost. Accounts of the trial made repeated reference to May Thorne’s benevolence in performing the operation free of charge. Miss Byrne was a housekeeper, suggesting that she was certainly not poverty-stricken, even though she saw Thorne on her ‘cheap days’. Private patients could also benefit from gratuitous treatment, as well as the privilege of recuperating in a nursing home, rather than a busy London hospital. A correspondent to *The Lancet*, Belfast doctor John Campbell, attacked what he saw as the unscrupulous nature of Miss Byrne in obtaining a ‘Free Operation’.^31^ By contrast, May Thorne had ‘operated without fee or reward, and has now been recompensed by the anxiety of a trial and the unpleasantness of a verdict against her’. Rather than attacking the usual working-class suspects, Campbell directed attention towards ‘well-to-do patients who have got operations done free in metropolitan hospitals or nursing homes and whose object in leaving their own localities was to escape the payment of surgical fees’. Indeed, from the account of Miss Byrne’s medical history given at the trial, she appeared to reside in Brighton, where the second operation to remove the sponge was performed. As Campbell noted, the more affluent classes were effectively abusing charitable systems designed to ‘benefit the deserving poor’. While Campbell was disgruntled primarily on behalf of ‘provincial surgeons’, whose patients sought metropolitan treatment, his letter seeks to direct attention away from any errors which might have been made by May Thorne and focus instead on the patient’s circumstances. Unfair to those less fortunate then herself, Miss Byrne is branded as undeserving and irresponsible.

## Risk

John Campbell’s letter pointed to another reason why patients might seek surgical assistance outside their own locality. In addition to the evasion of fees, there were simply more surgeons for patients to approach in the metropolis, all keen to advance themselves in specialist areas. ‘Gain[ing] experience’, as Campbell puts it, was vital to surgical advancement, as well as to the advance of surgery in the late nineteenth and early twentieth centuries. In the past few years, historians of medicine have also drawn attention to the 1890s and early 1900s as a period characterised by a distinctively experimental surgical outlook. This can be seen, on the one hand, in the sense of the scientific research, which reassuringly and firmly underpinned surgery at this point, and also, on the other, in the uncertainty created by new and untried procedures. Sally Wilde has written extensively about the surgeon ‘learning from mistakes’, progressing through experiments, performing major surgery which had never been attempted before.^32^ Wilde offers a fascinating conclusion about what she labels this ‘culture of innovation’; a creative process which saw confidence transferred between surgeons and patients about the possibilities of safe surgery through a ‘kind of groping towards circumstances under which surgery was justified, despite the very high risks’.^33^ Both Wilde and, more recently, Thomas Schlich, have echoed the comments made by Christopher Lawrence twenty years ago about the necessity of exploring the history of surgery through day-to-day practice or, as Schlich persuasively puts it in *The Origins of Organ Transplantation*, ‘[w]hat surgeons did in their jobs’, something which has become almost completely disregarded, obscured by the ‘social aspects’ of professionalisation.^34^ Examining specific cases where risk has resulted in a debate about responsibility, such as the May Thorne trial, allows further investigation into precisely how those who performed surgery during this period of experimentation considered ethical, as well as practical, issues both within and without the operating theatre.

May Thorne’s experience at the hands of a litigious patient and an unsympathetic judge, while detrimental to her character, did not harm her professional reputation. Indeed, it was confirmed and upheld by her fellow surgeons. It did, however, expose problems which might occur in the supposedly safe, early twentieth-century operating room, both on the table and between the personnel present. The Byrne versus Thorne trial had brought to the fore issues which had disrupted the new-found respectability and confidence of the surgeon. By 1904, thanks to the developments in surgical science during the second half of the nineteenth century, surgeons were no longer faced with the risks of having a conscious patient upon whom surgery had to be performed quickly and with brute, though skilful, force. While speed was no longer as essential or paramount as it had once been, surgical skill was still, of course, fundamental. Although the risks of surgery had decreased, correspondingly, surgical riskiness and the willingness of surgeons to take those risks had increased –to such an extent, indeed, in the 1890s and early 1900s, that both medical and lay outcry demanded that responsibility must be taken for actions. As May Thorne’s case reveals, an operation required the individual skill of the surgeon, coupled with the reliability and support of the surgical team. With increased personnel in the operating theatre, to administer anaesthesia or to assist the surgeon, responsibility for keeping the patient alive was shared amongst the team, as May Thorne’s defence counsel argued. However, the experimentation of the 1890s and 1900s witnessed, paradoxically, the promotion of the skilful surgeon over the team; glory was attributed to the individual, blame to the team. Success might be achieved, greater progress established through experimentation, but, claimed detractors, it was gained by risking responsibility.

## Surgical Anxieties

In May 1894 the *Daily Chronicle* newspaper ran a campaign to expose what it labelled ‘human vivisection’, a practice, it noted, which was occurring in hospitals throughout Britain.^35^ The outcry was part of a late Victorian obsession with the increasing power of medicine and the medical profession over defenceless individuals, who were stripped of their liberty by the probing instruments of scientific experimentation.^36^ Vaccinators and vivisectionists had borne the brunt of public loathing for over a decade: now it was the turn of the surgeon to be subjected to charges of brutality. ‘Houses of charity’, shrieked the *Chronicle*, were being turned, by younger, ambitious members of the surgical profession, into ‘butchers’ shops’, whereby innocent – and it was alleged, healthy – individuals were persuaded to undergo unnecessary and dangerous operations. Not for their own benefit, of course, but all for the desire to ‘destroy human lives in the interests of science’. According to the *Chronicle*’s exposé of hospital practice, the grasping surgeon experimented upon patients solely in order to keep up with the latest ‘surgical fads’, while unsuspecting patients simply agreed to the operator’s demands.

The paper lambasted the Dickensian youths who made up the new and future school of surgeons:

The new school consists of [. . .] enthusiasts who have only just passed from the stage at which young men go forth from the hospitals on football or boat-race nights to parade the West End in gangs [. . .]; and, having returned to their Bayswater or Bloomsbury lodging in the early morning and tried to sleep off the effects of bad whisky and worse cigars, go forth to gloat over men older than themselves destroying human lives in the interests of science.^37^

Louche, irresponsible, idle, and careless, these were the people in whose hands lay innocent lives: clumsy and dangerous youths, who could not be trusted. The claim that daring operative procedures represented progression was dismissed scornfully by the *Chronicle*, which branded contemporary surgery uncivilised and barbaric. In a celebratory edition, published for the Diamond Jubilee of Victoria’s accession to the throne, the *BMJ* begged to differ. Labelling the era a ‘Renaissance’ as far as the ‘advancement of surgery’ was concerned, the periodical concluded that ‘Heaven has given us a new race of men’. It was indeed a ‘Golden Age’:

The student sixty ago would see an occasional operation for strangulated hernia, perhaps an ovariotomy; there he would stop. The radical cure of hernia, known to Paré, had fallen into disuse; the surgery of the liver, the gall bladder, and the kidney, was unknown. Perforation of the stomach, or the bowels, or the appendix, was left to itself; cases of acute obstruction shared the same fate; so did ruptures of the abdominal viscera from external violence. The general work of abdominal surgery was hardly so much as attempted, save perhaps once or twice in a surgeon’s lifetime. Of such success as we there was not a trace.^38^

Whereas the *Chronicle* sees destruction and frailty, the *BMJ* envisages exploration and progress: the preservation of health rather than the wilful encouragement of illness. For the latter, the Victorian period had witnessed unprecedented ‘success’ through the development of procedures previously considered impossible and the conquering of disease in organs and parts of the body assumed inaccessible. Risk was essential to progress.

Concerns about an all-conquering surgical invasion were not just limited to popular newspapers with a non-too subtle stance on burning issues of the day. A thinly veiled fictional account written by one ‘Aesculapius Scalpel’, a disgruntled medical man, condemned the “*furor operativus*’, the operative madness [; that] burning desire to do everything that anybody had ever been known to do on the human subject in the way of surgery’. Especial attention was lavished on any case which would add lustre to the ‘most dangerous and difficult’ roll call of surgical procedures.^39^ At St Bernard’s, house surgeons compete to advance themselves through the performance of operations on the desperate and dying, who are given hope that some miracle can occur if only they submit themselves to the knife. The ‘live subjects’ are cajoled, bullied and bribed into exchanging their last moments for a procedure which could kill them more quickly, but which could make the name of their professionally climbing young surgeon. If the working-class patients are not cured through such wilful experimentation, then at least they will, in the future, benefit those of a higher class who, when it is their turn, are more likely to survive because of the sacrifice of their social inferiors.^40^ To ‘suffer so that surgeons might learn’ or to ‘die scientifically’ was, for the ‘case-hardened’ surgeons of this fictional hospital, a fitting dénouement for any patient.^41^ As an 1884 pamphlet entitled *Experiments on Patients* put it, Britain was in the grip of a socially demoralising ‘mania for Experimentation’, in the name of scientific research.^42^ Even animals had more legal protection.

The unclear distinction between practice and experimentation was also attacked in *St Bernard’s*. Anxious to defend the profession against accusations of ‘human vivisection’, medical periodicals both denied that experimentation was occurring and commended its practice. As the *BMJ* claimed in 1894, ‘operative practice can only be made by trying new methods, and that such trials [. . .] are the essential condition of surgical progress’.^43^ ‘Experimental experience’ was both ‘life-saving and conservative’.^44^ *St Bernard’s* condemns the hoodwinking of a patient who believes that an experienced surgeon will be skilfully performing an operation, carried out many times, upon himself. While it is ‘given out that the great man is to operate’ upon an injured workman, in reality, Dr Wilson, the house surgeon, anxious to contribute to a ‘trial of a new method of amputating the leg at the thigh’ carries out the procedure. Under anaesthetic, what patient could possibly know the difference? Here, the benefit of surgical progress – the anaesthesia – is manipulated to allow both for experimentation – the operation is a trial - and practice – so Dr Wilson can advance his career. As Senior Surgeon Bishop remarks after the operation: ‘I could have saved that leg if it had been my case, [. . .] but it would have been hard on Wilson to make him lose his chance’.^45^ Bishop’s use of ‘chance’ here is apt. At St Bernard’s, surgeons compete in a game to win the most accolades, as well as allow patients to undergo a double risk. Operated upon by the inexperienced Wilson, the man loses his leg, and his livelihood, in an unnecessary and experimental procedure. If surgeons advance in their profession through trial and error, Aesculapius Scalpel asks his readers to consider the unconscious and neglected patient in these proceedings. No wonder the (Irish, Catholic) workman can only eventually be convinced to gamble with his life by a priest.

While anti-vivisectionists and anti-vaccinationists brought the rights of hospital patients into their debates, surgeons themselves were considering carefully the wider implications of surgical advancement. Regular ‘Addresses in Surgery’, published in the medical press after society meetings, inaugurations or celebrations of the new academic year at universities, were increasingly sounding notes of caution as the nineteenth century ended. Preservation and conservation became watchwords indicative of successful surgery, which could and should be supported and cheered. The encouragement of the next generation was tempered with a warning about potential over-zealousness and an awareness of the scrutinising public gaze. Even confident reflections on the innumerable advantages of nineteenth-century progress were tempered with an anxiety about the future. Thomas Annandale’s 1898 ‘Address’ reviewed the countless benefits which scientific progress had brought to bear upon surgical practice; so far had Victorian surgery developed, indeed, that he was able to consider ‘the antiseptic system’ ‘almost [. . .] ancient history’. Although Annandale stressed that risk had been dramatically reduced for the patient because of the achievements of the past half century, intriguingly, he turns attention back to the surgeon, where the greatest riskiness is now firmly embodied. The ‘read[iness] to resort’ to surgery meant that the details of the case were often neglected in favour of instant operative results.^46^ When the risks of surgery were starkly evident, for the sake of both patient and reputation alike, even small, insignificant procedures had to be contemplated carefully. Annandale counselled that it would do no harm to consider, through a process of focused study, alternative options, before taking up the knife.

In 1903, the year before the Byrne versus Thorne case, Sir William Henry Bennett warned, in the oration given to the annual *Conversazione* of the Medical Society of London, about the contemporary disregard for ‘The Ethics of Operating’ and the all-too frequent readiness to ‘undertake operations without fully considering the ultimate advantage of their patient’. While Bennett was an advocate of the necessity of considering alternative methods of treatment, such as massage, his impressions of surgical practice were neither extreme nor rare:^47^

No operation could be considered absolutely safe, and the risk to life should be carefully weighed before deciding on the advisability of any operation. That a patient would certainly die if left alone was not in itself a sufficient justification of operation. The question that should guide the surgeon as to the amount of risk that could rightly be run was whether the lesion was due to curable disease or not. Of two operative procedures, by either of which the desired end might eventually be reached, the milder one should be preferred, even though less brilliant, or giving less obvious immediate results. Routine in operating was to be vehemently protested against – as applied to any particular disease it magnified its danger. Even operations involving no risk to life should not be undertaken without serious consideration. The interests of the patient, and not the mere attainment of a mechanical achievement, should be the first concern. Exploratory operations should not lightly be undertaken as a routine procedure on the plea that they would do no harm, even if no good resulted, for this was not always so, and in any case it was harmful in encouraging the neglect of extra-operative methods of diagnosis. Judgment was the enemy of routine, and routine was the bane of surgery. [N]o amount of anxiety on the part of a patient to undergo an operation could absolve a surgeon from responsibility as to its result. A too great regard for the good achieved by operations discounted the value of the preventive measures of surgical disease. Surgery was said to be a handicraft, but the knowledge of when to apply its craftsmanship was of the first importance.^48^

The brilliance of showmanship, coupled with the addiction of success, ensured that experimentation had paradoxically become routine. As surgery became correspondingly ‘safer’, risks had increased and the patient’s condition subsumed under a desire for glorified irresponsibility. For William Henry Bennett, theory and practice should be supported by an ethical consideration of the benefits to be accrued to the patient who undergoes the procedure and not simply the skilfulness of the achievement.

Yet Bennett’s diatribe against those all too eager to cut open their patients was aimed not only at the surgeons themselves. Within his speech was a brief sentence which suggested that risk-taking was not one-sided: patient choice was also a factor. ‘The dread of necessary operations by patients’, noted Bennett, ‘was in marked contrast to their craving for inexpedient and unnecessary operations’.^49^  If the late nineteenth and early twentieth centuries were characterised by surgical experimentation, it was not simply the surgeons themselves who drove the demand for increasingly dangerous procedures. In the final sections of this article, I will explore what can be labelled patient perversity, focusing on women patients more generally, as well as specifically, at the female-run New Hospital for Women.

## Patient Perversity

The increased ‘safety’ of surgery in the latter part of the nineteenth century and the effect of anaesthesia had effectively removed the troublesome aspect of a conscious patient from operations. However, lack of consciousness could prove just as worrying for the patient, not only because, as was seen in St Bernard’s, anyone could be carrying out the operation, but an anaesthetised patient could not consent to additional procedures if difficulties or problems were encountered during surgery. The fear of death under anaesthesia could also prevent some patients from agreeing to surgery in the first place, however serious their condition. Individual concerns were also fuelled by articles in the popular press about the dangers of anaesthesia.^50^ For some prospective patients, one form of terrifying risk had simply been exchanged for another. Fears about anaesthesia, coupled with publicity in the 1890s over ‘human vivisection’, ensured that surgeons were, as Clifford Hawkins claims, ‘more liable than physicians to litigation’.^51^ If patients were litigious, they were simultaneously perverse in their demands. As Lawson Tait noted, the *fin-de-siècle* surgeon ‘runs risk of action at law on the one hand for removing healthy organs, and on the other hand of being abused for not removing organs in which no trace of disease existed’.^52^ Patients’ bizarre or inappropriate demands fuelled suspicion of surgical necessity. Those carrying out abdominal surgery, especially upon women, were particular targets for patient distrust and public scorn. The popular misconception that female patients would be ‘unsexed’ by any abdominal procedure was one which fuelled attacks upon women’s surgeons, especially at a time when a high rate of infant mortality occupied social anxieties.^53^ One lengthy and distressing legal case embodies a number of these fears, as well as leading, in 1897, to the formation of the Society for the Protection of Hospital Patients: Beatty versus Cullingworth.

In similar fashion to the May Thorne trial, the repeated attempts of hospital nurse Alice Beatty to take her surgeon, Dr Charles Cullingworth, to court encouraged professional concerns about litigious patients. Claiming that Cullingworth had operated upon her without consent, Beatty also alleged assault.^54^  Her accusation centred on the fact that Cullingworth had removed both her ovaries, despite only intending to operate upon her right, prolapsed one. This was something which she had made clear from the outset that she would not accept, as she had wanted to marry and have children. Cullingworth’s actions had now ensured the latter was impossible. He claimed that the operation was necessary and that he had acted with her full consent when, under chloroform, he discovered that the condition of the left ovary matched that of the right, and both were removed. Unlike the May Thorne case, Cullingworth’s medical witnesses did not wholly agree with the surgeon’s judgement. Despite never having examined the patient, Spencer Wells and Bedford Fenwick both felt that Cullingworth ought not to have removed the left ovary if an express desire had been articulated by Miss Beatty to avoid this at all costs. As the latter noted, if a problem had been discovered then it should have been mentioned to Miss Beatty when she awoke from the anaesthesia, allowing her to make a decision based upon detailed surgical knowledge of her condition. Fenwick, however, highlighted simultaneously the lack of concern for patient opinion when he questioned the difference between a ‘wish’ and an ‘order’. This revealed, of course, that the patient was in no position to command their surgeon.^55^ Again, as in the case of May Thorne, Cullingworth’s ‘skill’ was mentioned numerous times during the trial, as well as his life-saving thoughtfulness. While the operation had been completed four years before, in 1892, Alice Beatty had been persecuting Cullingworth ever since with actions against him and her ‘mental excitability’, and that of her female supporters, was frequently noted. Cullingworth’s defence counsel argued that he had informed Miss Beatty from the outset that his judgement must be trusted, but he could give no guarantee that only one ovary was affected. Only he could know and judge when he opened her up.

Cullingworth was clear that he ‘accepted the responsibility’ for the operation and would not consult with the patient’s sister, who was waiting in another room, because she could not possibly make an informed clinical judgement about the risk of leaving the left ovary.^56^ Here, Cullingworth embraced both risk and responsibility. But, for Alice Beatty, he had ‘overstepped’ his position, disregarding her clearly stated opinion, rendered her infertile and, consequently, assaulted her under anaesthetic. Throughout the trial, it became apparent that patient and surgeon were not communicating with each other during the period of consultation. While Beatty insisted that her left ovary be undisturbed, regardless of its condition, Cullingworth, refusing to guess at the outcome of the operation, stated that the patient must trust the surgeon. Cullingworth was keen to stress that the patient’s interests and future health had been always at the forefront of his mind. He had not performed the procedure to profit financially – he had operated *gratis* – nor had he sought to benefit professionally – he had neither written about, lectured upon, nor exhibited Miss Beatty’s ovaries, which had been destroyed. A further medical witness, Dr Herman, concluded that Cullingworth could not have ‘unsexed’ Alice Beatty, because, if her ovaries were as described in her surgeon’s notes, then she would most likely have been sterile before the operation commenced and dead within a decade if they had not been removed. Lawson Tait concurred, noting that the patient’s health and been restored and her life prolonged by Cullingworth’s surgical risks. Judge and jury agreed, condemning Alice Beatty for bringing the action in the first place. Tacit consent had been provided and the patient had tried effectively to ‘fetter’ her surgeon by not allowing him to take charge of the situation.^57^

Alice Beatty appealed against the decision, but her plea was dismissed out of hand, without the defence’s evidence being heard.^58^ With some supporters, Beatty then established and became Secretary of the Society for the Protection of Hospital Patients in early 1897. This organisation was ‘formed to protest against the growing tendency among the medical profession to regard the hospitals as endowed schools for experimental research, and to assist patients in asserting their legal rights as against unwarranted operations and otherwise’.^59^ An offshoot of societies which sought to defend animals and humans alike from the exploitation of scientific and medical experimentation, Beatty’s organisation received similar mixed press. *Reynolds’s Newspaper* mocked the society as a ‘bevy of lady enthusiasts’, who were concerned with the depopulation of society because of the increase in surgical ‘unsexing’.^60^ Similarly, *Lloyd’s Weekly Newspaper* questioned the society’s foundations by designating it dubious, with a ‘so-called’ status.^61^ The *Woman’s Signal*, however, looked upon the new organisation with a more sympathetic, if guarded tone. Without commenting on the precise details of the Beatty case, the periodical considered the wider implications. Troublingly, it concluded, this case implied that ‘a surgeon may do exactly what he pleases on a patient insensible under an anaesthetic, and need only swear that he held his action to be good for the patient’.^62^ In other words, the surgeon need take no responsibility for risky actions, providing they were always intended to improve the patient’s situation. Any individual, desperate for the restoration of health, could be easily persuaded to undergo a procedure if it meant an end to their suffering. For the *Woman’s Signal*, the most susceptible and easily convinced patients would be the poor who, without scientific understanding or the means to pay either for the procedure in the first place or redress if anything went wrong, were vulnerable to exploitation. While Alice Beatty was not impoverished, she had not paid for her operation and the *Woman’s Signal* linked her case directly with those of patients who could not pay and who, of course, would have their operations carried out in hospitals.

In September 1897 a correspondent to *Reynolds’s Newspaper*, in a letter entitled ‘Hospital Scandals’, reiterated this point. ‘Northumbrian’ commented that the country’s hospitals were profiting enormously from charitable impulses connected with Queen Victoria’s Diamond Jubilee, but the same hospitals were also peopled with those who were ‘callous to human suffering’. The hospital surgeon, claimed ‘Northumbrian’, in a familiar refrain, ‘excuses all his experiments on the ground that he is doing something for the benefit of the human race generally’. ‘But’, concluded the correspondent, ‘you never find him trying these experiments upon his paying patients’.^63^ The surgical scandals of the 1890s and early 1900s affected one type of establishment more than any other: women’s hospitals. As a disillusioned member of the profession, Edward Berdoe, claimed: ‘hospitals for women are the happy hunting ground for the human vivisector’.^64^ Not only were women’s hospitals frequently charitable institutions where patients paid little if anything for treatment, they were also home to that most ‘murderous’ and ‘belly-ripping’ of practitioners: the abdominal surgeon.^65^ When the Chelsea Hospital for Women came under public scrutiny in 1894 for what was seen as a disproportionate number of fatal operations, detractors were swift to assign blame to over-zealous surgical personnel, even though poor hygienic conditions caused by the drains were actually found to be responsible.^66^ Women like Alice Beatty or patients at the Chelsea were simply hoodwinked into believing their symptoms would be alleviated through unnecessary surgical intervention. While vehemently defending abdominal surgeons, even the *BMJ* found difficulty in labelling ‘novel operations’ for uterine conditions either life-saving or offering more than ‘fair prospects’. If women were prepared to ‘run some risk’ to avoid disability, however, dangers could be justifiable.^67^ Here, rather than the exploitation of the patient by the surgeon, the *BMJ* places responsibility for the decision to undergo difficult and dangerous surgery with the former.

## Risk and the Woman Surgeon

The *Daily Chronicle* sought to add another level of controversy to the debate over ‘human vivisection’ at women’s hospitals when it consulted Elizabeth Blackwell. Blackwell was the *grande dame* of women doctors and, at the age of seventy-three, had a great deal to say about the alarming, as she saw it, trajectory of the woman who performed daring surgical procedures. Blackwell feared that surgery, with its glamorous, radical and daring status, had replaced medicine as a cure-all. The modern day approach, she felt, was too hurried, too impatient, too willing to open up a patient before fully ascertaining what was really wrong with them. Women were particularly the victims of this too ready recourse to the knife. Blackwell’s concerns were not only for helpless women patients, however. She used the *Chronicle* reporter to voice her distrust of those women only too keen to perform ‘reckless operations’ which ‘maim[ed]’ their own sex for life. Prompted by what sounded like an attack upon her fellow medical women, the reporter asked: ‘Do you consider that women practitioners are less liable to this “operative madness” than men?’ Blackwell’s response was intriguing:

I have no hesitation in saying that *at present* my own sex is suffering from the epidemic, but it is imparted to them by their surroundings. You see it is very contagious. They learn from men, and live in the atmosphere of surgery. They are over-anxious to do as men do, and so their reverence of creation and their sympathy for the poor and suffering is in abeyance. A woman – and a very clever one – boasted recently that she had just completed her fiftieth operation of a particular and very dangerous kind – a kind such as I think it must have been difficult to find within her reach, fifty cases in which it was necessitated. She had probably been taught that the operation was frequently necessary, and she is no more reckless than those who taught her; but her sense of humanity was, perhaps, for a while in abeyance. I am, however, persuaded that this will pass away so far as women are concerned; the danger is an almost inevitable accompaniment of the early stages of a movement in the ultimate success of which I have the greatest belief – the educating of women so that they may alleviate the physical sufferings of their own sex, not only as nurses, but as physicians and surgeons. I do not believe that the study and practice of surgery necessarily tend to unsex a woman. It is a noble work, this curing of disease, and must be nobly done, whether by men or women.^68^

When younger, Blackwell herself had intended to devote her life to surgery, but had been infected with purulent ophthalmia by a young patient at La Maternité in Paris, which had left her blind in one eye, incapable of intricate surgical procedures and unable to become, as she had hoped, ‘the first lady surgeon in the world’.^69^ So, while Blackwell did not believe that the existence of the woman surgeon was wrong per se, the aping of the infectious masculine swagger of surgical glory could only detract from the true ‘curing of disease’. It was this discrepancy between the risks involved in opening up a patient and the ultimate restoration of health which was so distressing for the elderly pioneer medical woman.

It is also noticeable that Blackwell draws attention to a clever female boaster, who can only be Elizabeth Garrett Anderson, whose daring procedures were carried out at the New Hospital for Women, where women formed the medical and surgical staff. Indeed, even though Blackwell was still acting – in name if not literally – as a consultant physician there, the management committee of the hospital chose to maintain a dignified silence over these pointed accusations. In the first meeting of the committee after the article was published, on 31 May 1894, the notes record that the Chairman, Mr Gaselee, will write to Garrett Anderson to prevent her from responding to Blackwell’s article or being interviewed in the *Daily Chronicle*.^70^ The New Hospital was still undergoing its own decade-long internal divisions over surgical procedures and to draw attention to the painful schisms which had existed in the hospital would not encourage its patients to undergo some of the operations at which Elizabeth Blackwell was hinting.^71^ While Blackwell was clearly willing, anonymously, but evidently, to critique the hospital for which she still acted as consultant, that hospital was not willing to involve itself in an unseemly debate which could only sorely inflame public sensibilities and hint that all was not well among the ‘lady doctors’ there.

After internal controversies over surgical risk-taking by Garrett Anderson in the late 1880s, which had led to the resignation of a number of members of staff and eventually Garrett Anderson’s own departure, the next decade was very different. From 1892 the team of Mary Scharlieb and Florence (better known as Mrs Stanley) Boyd took over the surgical side of the hospital. With the change in personnel, came an alteration in the rules of operation. The Minutes of the Management Committee of the Hospital record in December 1892, after Garrett Anderson’s departure, the requirement that surgery can only be carried out by those experienced enough to do so.^72^  Experience here counted entirely: no one could perform abdominal operations unless they had assisted at a dozen procedures at the hospital. Exactly two years later, and nearly seven months after the *Chronicle* article, Mrs Scharlieb and Mrs Boyd were honoured with the precise title of ‘surgeon’.^73^  Rather than wavering in its performance of risky and controversial operations after the concerns raised about procedures at the hospital, the competence of its staff and the growing public worries about the wisdom of surgery, the New Hospital for Women determined to prove its success in promoting the woman surgeon and defending her actions.

The *Annual Reports* for the 1890s illustrate the growth of risky operations, including bladder, gastric, kidney, liver and rectal procedures, as well as for the diseases of women. While the New Hospital catered for women and children, it did not consider itself a specialist institution and always insisted on its status as a general hospital, reflected here in the number of different procedures performed.^74^ In the first year of Scharlieb’s promotion to senior surgeon, 1893, operation deaths were at their highest ever: eight out of fifty-one major cases; for three years, between 1894, 1895 and 1897, there were five deaths out of fifty-nine, seventy-two and ninety-three operations, respectively; in 1896 there was only a single death out of seventy-four operations; in 1898 three out of eighty-seven; and in 1899 the number had risen to seven out of 118 cases. From 1900 there is a distinct correlation between the number of deaths and the number of patient refusals. In 1900 there were eight operation deaths and nine refusals out of 130 cases; in 1901 ten deaths and ten refusals, from a total of 149 cases and, in 1902, eight operation deaths and seven refusals in 155 major operation cases.^75^ In 1902 Mary Scharlieb took a coveted specialist post as surgeon for women’s diseases at the Royal Free Hospital – the first woman to hold such a senior position in a general institution.^76^ By the time she left, there were three times as many major operations carried out at the New Hospital for Women as there had been ten years earlier, but with the same number of fatalities: an impressively consistent statistic. The growing confidence of Mary Scharlieb as a surgeon contributed to an exponential increase in risky operative procedures, but a steadying hand in controlling surgical and post-surgical death.^77^ Typically self-effacing about her abilities, Scharlieb herself attributed survival rates partly to the development of pathology at the hospital, which allowed the surgeon greater accuracy of diagnosis and treatment.^78^

What we see alongside this, however, is a growth in patient complaint and resistance, suggesting that the patient herself was far more willing by the early Edwardian period than her Victorian equivalent to refuse operative interference, despite the increasing ‘safety’ of procedures. Fundamental here to note is that patient refusal has very little to do with the statistical riskiness of the operation and, indeed, there is no consistent pattern in the kinds of procedures patients will not undergo. In the three years between 1900 and 1902 there were twenty-six refusals of surgical treatment for various ailments out of 434 major operation cases, or just under six per cent of the total number. Situations where operations were actually carried out for the same conditions reveal that there were only five deaths: two for fibroids; two for tubercular peritonitis; and one for cancer of the uterine body. Over the three years, successful operations numbered thirty-four, three and six respectively.^79^ What the patient perceived as a risk-filled undertaking was, on the whole, statistically unlikely to be the sort of operation where death rather than cure or relief resulted. Surgical success, therefore, had very little to do with patient perception of the operation itself.

Patient perception of a cure, however, was one area where radical and conservative surgery met and where the woman surgeon could defend her decision to operate. In a fascinating and strikingly modern follow-up of patients from the New Hospital for Women, published in the *BMJ* in 1899, May Thorne, assistant anaesthetist, former senior house surgeon to the New Hospital and later at the centre of the Byrne negligence trial, interviewed ex-patients to discover the post-operative effect of an abdominal section on the patient’s mentality and lifestyle.^80^ Ostensibly responding to a paper given by Herbert Spencer at the Obstetrical Society in 1897, which explored complications after initial surgery requiring further procedures, Thorne explored the after-history of eighty-eight patients from the New Hospital between the key years of 1888 and 1897. Of these eighty-eight, only three had returned for further surgery; one of these had died after being re-admitted in a ‘moribund’ condition, without a further operation. Comprising precisely the operations considered to unsex women, such as removal of the ovary or ovaries, this list offers an intriguing glimpse into how female patients viewed operations which might be labelled ‘human vivisection’. While the questions asked clearly suggest the restoration of health rather than suffering, the answers are certainly not uniform and many are quoted verbatim, allowing a brief glance at the individual, though anonymous, respondent. Of the eighty-two surviving patients who had not returned for further procedures or died of other causes, only ten described their health as poor or ‘not good’, with just under half of these recurring symptoms probably related to the causes for which they originally underwent an operation. Three mentioned resuming or beginning occupations with a differing variety of physical labour required, from mangling to managing a busy hotel, or working for the Salvation Army. Three either had or were going to have a child since their procedure, scotching the popular misconception that any form of abdominal surgery upon women resulted in sterility. Nearly a quarter claimed excellent health, and a quarter mentioned the operation itself leading to an improvement in their condition. Indeed, every single one of those who did discuss the operation claimed improvement. What is clear is that, even though this must have been presented as a leading question, asked along the lines of, ‘how is your health since the operation?’, those who enjoyed good or better health attributed their recovery to the surgical procedure and those who still suffered did not suggest that it was a cause in their continued ill health. The ultimate result, of course, is a magnificent vindication of the carrying out of operations on women by women. Such an article was something which could certainly bolster the New Hospital’s perception of its surgical success in the past decade, despite its own internal obstructions.

## Risk, Responsibility, Surgery

Risk, therefore, was contingent upon its perception as well as its practise. Even with the apparent elimination of the dangers inherent in surgery without antisepsis or asepsis, the correspondingly ‘safer surgery’ became riskier. While Sally Wilde has suggested that with surgical came patient confidence, the latter did not necessarily perceive risk in the same way as the former.^81^ In the 1890s and early 1900s surgeons were scrutinised very carefully by their prospective patients. As Lawson Tait lamented: ‘in these days [. . .] we have to run the risk of hearing our surgical judgments overhauled by a jury of inquiry, and our surgical operations condemned in the witness box by nurses’.^82^ Patient perversity was a contributory factor, Tait concluded, to the era’s contradictory attitude towards surgery. It was this fear of increasing publicity, especially concerning cases of surgical paraphernalia being left inside the body, which caused the principal ‘harassing dread’ for early twentieth-century surgeons, according to the *BMJ*, a month before May Thorne’s case came to trial in 1904. ‘Responsibility’, it concluded, ‘must always be to a certain extent divided’ amongst all the surgical personnel. The argument here is finalised, however, with a glance around the hierarchy, rather than the democracy, of the operating theatre: ‘The operator fatigued by an emergency operation, which he has just concluded, is often not in a fit state to make sure about forceps and sponges without the intelligent aid of his assistants and nurses’.^83^ For the surgeon, now supported in the early twentieth century by a whole team, riskiness could be controlled by the removal of focus upon individual surgical skill. Yet it could also, as in the case of the New Hospital for Women and even, most incongruously, in the May Thorne negligence trial, be used to promote skilful surgical achievement.

## Footnotes

1. Accounts of the Thorne trial, from which specific details are taken, can be found in ‘Medico-Legal and Medico-Ethical: An Overlooked Sponge’, *BMJ*, 1, 2267 (11 June 1904), 1408–9; ‘Medicine and the Law: Byrne v Thorne’, *The Lancet*, 164, 4223 (6 August 1904), 419–20; ‘The Law Courts’, *The Times*, 37414 (Tuesday 7 June 1904), 9.

2. SchlichThomas, ‘Risk and medical innovation: A historical perspective’, in Thomas Schlich and Ulrich Tröhler (eds), The Risks of Medical Innovation: Risk Perception and Assessment in Historical Context (Abingdon and New York: Routledge, 2006), 1–19: 16.

3. See, for example, *Twenty-Ninth (1900) Annual Report of the New Hospital for Women, 144, Euston Road, N.W. Established 1872* (London: Alexander and Shepheard, 1901), 6, where Thorne appears as a ‘Senior Assistant’ and ‘Anaesthetist’.

4. Thomas Annandale, ‘Address in Surgery’, *BMJ*, 2, 1961 (30 July 1898), 293–7: 295; ‘Count Sponges and Instruments’, *BMJ*, 1, 2052 (28 April 1900), 1047–8.

5. Christopher Lawrence, ‘Democratic, divine and heroic: the history and historiography of surgery’, in C. Lawrence (ed.), *Medical Theory, Surgical Practice: Studies in the History of Surgery* (London and New York: Routledge, 1992), 1–47: 7; 32. See also Sally Wilde and Geoffrey Hirst, ‘Learning from Mistakes: Early Twentieth Century Surgical Practice’, *Journal of the History of Medicine and Allied Sciences*, 64, 1 (2008), 38–77; L.S. Jacyna, ‘The Laboratory and the Clinic: The Impact of Pathology on Surgical Diagnosis in the Glasgow Western Infirmary, 1875–1910’, *Bulletin of the History of Medicine*, 62, 3 (1988), 384–406.3067788

6. Sally Wilde concludes that, by 1900, the possibility of surgical confidence had been passed to the patient, while Ian Burney notes that there was a ‘confident, professionalised interaction among surgeon, patient and anaesthetist’, which was disrupted by the publicity given to anaesthetic deaths. See Sally Wilde, ‘Truth, Trust, and Confidence in Surgery, 1890–1910: Patient Autonomy, Communication, and Consent’, *Bulletin of the History of Medicine*, 83, 2 (2009), 302–30; 305: 328; and Ian Burney, *Bodies of Evidence: Medicine and the Politics of the English Inquest*, *1830–1926* (Baltimore, MD and London: Johns Hopkins University Press, 2000), 153.

7. See note 1 for sources.

8. *BMJ*, *op. cit.* (note 1), 1409.

9. *BMJ*, *op. cit.* (note 1), 1409.

10. *The Times*, *op. cit.* (note 1).

11. Frederick Treves, *Manual of Operative Surgery*, Vol. II (London: Cassell & Company, 1892), 234.

12. *Lancet*, *op. cit.* (note 1), 420.

13. *Lancet*, *op. cit.* (note 1), 420.

14. For a history of the Medical Defence Union, see Clifford Hawkins, *Mishap or Malpractice?* (Oxford: Blackwell/Medical Defence Union, 1985).

15. See *BMJ*, 2, 2271 (9 July 1904), 100; *The Lancet*, 164, 4219 (9 July 1904), 122.

16. ‘Correspondence: Thorne Defence Fund’, *BMJ*, 1,2268 (18 June 1904), 1462; also in *The Lancet*, 163, 4216 (18 June 1904), 1750.

17. ‘The Responsibility of a Surgeon for His Assistants’, *BMJ*, 1, 2268 (18 June 1904), 1446–7: 1446.

18. *Ibid*, 1447.

19. *BMJ*, *op.cit.* (note 17).

20. Printed in *BMJ*, 2, 2272 (16 July 1904), 153; and *The Lancet*, 2, 4220 (16 July 1904), 173.

21. ‘Room for the Ladies!’, *BMJ*, 1, 119 (9 April 1859), 292-4: 293.

22. Thomas Schlich, ‘Surgery, Science and Modernity: Operating Rooms and Laboratories as Spaces of Control’, *History of Science*, 45 (2007), 231–56: 236.

23. Stefan Hirschauer, ‘The Manufacture of Bodies in Surgery’, *Social Studies of Science*, 21, 2 (1991), 279–319: 297.

24. Treves, *op. cit.* (note 11), 237.

25. Treves, *op. cit* (note 11), Vol. I, 36–7.

26. Ghislaine Lawrence, ‘The ambiguous artifact: surgical instruments and the surgical past’, in Christopher Lawrence (ed.), *op. cit.* (note 5), 295–314: 310–11.

27. Treves, *op. cit.* (note 11), 29. This is not to suggest that Treves’s statement is contradictory; rather, the ‘brilliance’ of early nineteenth-century speed and *sang froid* have been replaced, in his eyes, by the splendour of intricacy, precision and the possibility of replication.

28. A. Marmaduke Sheild, ‘Sponges and Forceps Left in the Abdominal Cavity’, *The Lancet*, 163, 4215 (11 June 1904), 1684. All quotations in this paragraph are from the letter, which was dated 6 June 1904, the final day of the Thorne trial.

29. *The Lancet*, *op. cit.* (note 1), 419–20: 417. For ongoing tensions between standardisation and individual surgical skill, see Thomas Schlich, ‘The Art and Science of Surgery: Innovation and Concepts of Medical Practice in Operative Fracture Care, 1960s–1970s’, *Science, Technology, & Human Values*, 32, 1 (2007), 65–87.

30. Keir Waddington, ‘Unsuitable Cases: The Debate over Outpatient Admissions, the Medical Profession and late-Victorian London Hospitals’, *Medical History*, 42 (1998), 26–46: 29.10.1017/s0025727300063328PMC10439679536622

31. Letter from John Campbell, dated 8 August, 1904, ‘Free Operations’, *The Lancet*, 164, 4224 (13 August 1904), 491. All quotations in this paragraph are from this letter.

32. See, for example, Sally Wilde, *The History of Surgery: Trust, Patient Autonomy, Medical Dominance and Australian Surgery, 1890–1940* (e-book at: www.thehistoryofsurgery.com); Wilde, *op. cit.* (note 6); Wilde and Hirst, *op. cit.* (note 5); Sally Wilde, ‘The Elephants in the Doctor–Patient Relationship: Patients’ Clinical Interactions and the Changing Surgical Landscape of the 1890s’, *Health and History*, 9, 1 (2007), 2–27.

33. Wilde, *op. cit.* (note 6), 219.

34. Thomas Schlich, *The Origins of Organ Transplantation: Surgery and Laboratory Science, 1880–1930* (Rochester, NY: University of Rochester Press, 2010), 9–10; Lawrence, *op. cit.* (note 5), especially 10–15.

35. Richard D. French notes that the *Daily Chronicle* was one of the most consistent backers of the anti-vivisection movement. See *Antivivisection and Medical Science in Victorian Society* (Princeton, NJ: Princeton University Press, 1975), 267.

36. For an account of the reaction against medical intervention during this period, see, for example, Nadia Durbach, *Bodily Matters: The Anti-Vaccination Movement in England, 1853–1907* (Durham, NC and London: Duke University Press, 2005).

37. ‘The *Daily Chronicle* on Human Vivisection’, *BMJ*, 1, 1743 (26 May 1894), 1143–4: 1143.

38. ‘The Renaissance of Surgery in the Victorian Age: Abdominal Surgery, 1837–1897’, *Queen’s Commemoration Number, BMJ*, 1, 1903 (19 June 1897); 1527–31: 1527; 1530.

39. Aesculapius Scalpel [Edward Berdoe], *St Bernard’s. The Romance of a Medical Student* (London: Swan Sonnenschein & Co., 1888), 88.

40. *Ibid*, 61.

41. Scalpel, *op. cit.* (note 39), 92. ‘Dying Scientifically’ was an alternative title for what appears to have been a pamphlet form of the novel, published by the Society for the Protection of Hospital Patients. See ‘Hospital Scandals’, *Reynolds’s Newspaper*, 2456 (Sunday 5 September 1897), 2.

42. Anon., *Experiments on Patients by Two Hospital Physicians*, 2nd edn, with additions (London: Pickering & Co., 1884), 17.

43. ‘The Chelsea Hospital for Women’, *BMJ*, 2, 1756 (25 August 1894), 429-36: 431.

44. ‘Experiments on Patients’, *BMJ*, 1, 1745 (9 June 1894), 1262.

45. Scalpel, *op. cit.* (note 39), 88-9: 92.

46. Annandale, *op. cit.* (note 4), 293-7: 294; 295–6. For the context of Annandale’s comments about ‘historical’ antisepsis and the rise of asepsis in the 1890s, see Michael Worboys, *Spreading Germs: Disease Theories and Medical Practice in Britain, 1865–1900* (Cambridge: Cambridge University Press, 2000), 150–92.

47. ‘Obituary: Sir William Bennett, KCVO, FRCS’, *BMJ*, 1, 3705 (9 January 1932), 80–1: 81.PMC251997420776610

48. Sir William Henry Bennett, ‘The Ethics of Operating’, *BMJ*, 1, 2212 (23 May 1903), 1222. See David S. Jones, ‘Visions of a Cure: Visualization, Clinical Trials and Controversies in Cardiac Therapeutics, 1968–1998’, *Isis*, 91, 3 (Sept 2000), 504–41 for a more recent example of professional differences over the interpretation of surgical success. For the performance of surgical identity, see Delia Gavrus, ‘Men of Dreams and Men of Action: Neurologists, Neurosurgeons, and the Performance of Professional Identity, 1920–1950’, *Bulletin of the History of Medicine*, 85, 1 (2011), 57–92.

49. Bennett, *op. cit.* (note 48).

50. For more on late nineteenth century fears of anaesthetic death, see Stephanie J. Snow, *Operations Without Pain: The Practice and Science of Anaesthesia in Victorian Britain* (Basingstoke: Palgrave Macmillan, 2006) and Burney, *op. cit.* (note 6) and ‘Anaesthesia and the evaluation of surgical risk in mid-nineteenth-century Britain’, in Schlich and Tröhler (eds), *op. cit.* (note 2), 38–52.

51. Hawkins, *op. cit.* (note 14), 12. Hawkins suggests that this is because surgical negligence could be ‘seen, such as the failure of a wound to heal, whereas physicians’ mistakes could easily be attributed to nature’.

52. Lawson Tait, ‘The Modern Treatment of Myomatous Disease of the Uterus’, *BMJ*, 1,1891 (27 March 1897), 779–80 780.10.1136/bmj.1.1891.779PMC243294720756887

53. See, for example, Anna Davin, ‘Imperialism and Motherhood’, *History Workshop Journal*, 5 (1978), 9–65.10.1093/hwj/5.1.911610463

54. The following account of the case is taken from ‘Beatty v Cullingworth’, *BMJ*, 2, 1873 (21 November 1896), 1525–6 and ‘Medico-Legal. Beatty v Cullingworth: *Times* Report’, *ibid.*, 1546–8.

55. ‘Medico-Legal. Beatty v Cullingworth’, *op. cit.* (note 54), 1547.

56. Medico-Legal. Beatty v Cullingworth’, *op. cit.* (note 54), 1548.

57. Medico-Legal. Beatty v Cullingworth’, *op. cit.* (note 54). Beatty would later publish two books which stemmed from her encounter with Dr Cullingworth: *Unjust Judges* (London: Judicial Injustice Redress Society, 1911) and the self-published *Medical Tyranny: A Personal Experience* (London: A.J. Beatty, 1912).

58. ‘Medico-Legal: Beatty v Cullingworth’, *BMJ*, 1, 1881 (16 Jan 1897), 178.

59. ‘London Week by Week: Miscellaneous’, *Reynolds’s Newspaper*, 2426 (Sunday 7 February 1897), 1.

60. ‘Notes and Gossip’, *Reynolds’s Newspaper*, 2423 (Sunday 17 January 1897), 1.

61. ‘Sunday’s Survey’, *Lloyd’s Weekly Newspaper*, 2844 (Sunday 23 May 1897), 10.

62. ‘Signals from Our Watch Tower’, *Woman’s Signal*, 165 (Thursday 25 February 1897), 120.

63. ’Hospital Scandals’, *Reynolds’s Newspaper*, 2456 (Sunday 5 September 1897), 2.

64. ‘The Chelsea Hospital for Women’, *BMJ*, 2, 1756 (25 August 1894), 429–36: 431. Edward Berdoe would later become ‘Aesculapius Scalpel’ and was responsible for the *Daily Chronicle* piece on ‘Human Vivisection’ quoted in this article.

65. *Ibid*

66. For more on this scandal, see Ornella Moscucci, *The Science of Woman: Gynaecology and Gender in England, 1800–1929* (Cambridge: Cambridge University Press, 1990), 92–101.

67. *BMJ*, *op. cit.* (note 61), 431.

68. ‘Human Vivisection: Interview with Dr Elizabeth Blackwell’, *Daily Chronicle*, 22 May 1894, Newspaper Cuttings: New Hospital for Women, 1871–1968, H13/EGA/144 (London Metropolitan Archives).

69. BlackwellDr Elizabeth, Pioneer Work in Opening the Medical Profession to Women. Autobiographical Sketches (London and New York: Longmans, Green and Co, 1895), 154; 157.

70. *Minute Books of the Management Committee of the New Hospital for Women, II: May 1882–March 1895*, H13/EGA/20 (London Metropolitan Archives).

71. See Claire Brock, ‘Surgical Controversy at the New Hospital for Women, 1872–1892’, *Social History of Medicine*, 24, 3 (December 2011), 608–23.

72. *Minute Books*, *op. cit.* (note 70), 1 December 1892.

73. *Ibid*, 6 December 1894.

74. See, for example, A. Gordon Pollock’s letter to the Hon. Secretaries of King Edward’s Hospital Fund, dated 27 July 1905, where he states that the hospital is not one solely for the ‘special diseases of women’, but a ‘general Hospital for women desiring to be attended by physicians and surgeons of their own sex’, King Edward’s Hospital Fund for London Correspondence with the New Hospital for Women, A/KE/248/4 (London Metropolitan Archives).

75. See *Annual Reports of the New Hospital for Women* (London: Alexander and Shepheard), published between 1894 and 1903.

76. ScharliebMary, Reminiscences (London: Williams and Norgate, 1924), 167–169

77. See, for example, Scharlieb’s article ‘Surgery at the New Hospital for Women in 1896’, *BMJ*, 2, 1910 (7 August 1897), 338–9.10.1136/bmj.2.1910.338PMC240744920757201

78. *Ibid*, 338, where pathologist’s reports prevent the carrying out of a wrong diagnosis into surgical errors.

79. See *Annual Reports of the New Hospital for Women* between 1901 and 1903.

80. May Thorne, ‘After-Effects of Abdominal Section’, *BMJ*, 1, 1988 (4 February 1899), 264–5. Even nearly a decade later, following up patients, either by letter or in person, was only carried in post-operative cancer cases at the New Hospital. See Louisa Garrett Anderson and Kate Platt, ‘Malignant Disease of the Uterus. A Digest of 265 Cases Treated in the New Hospital for Women’, *Journal of Obstetrics and Gynaecology of the British Empire*, 14, 6 (December 1908), 381–92: 381.

81. Wilde, *op. cit.* (note 6), 328.

82. Tait, *op. cit.* (note 51), 780.

83. ‘Overlooked Forceps’, *BMJ*, 1, 2263 (19 May 1904), 1147.

